# Prognostic value of structural variants in early breast cancer patients

**DOI:** 10.1038/s41523-024-00669-9

**Published:** 2024-07-27

**Authors:** Ji-Yeon Kim, Kyunghee Park, Woong-Yang Park, Jin Seok Ahn, Young-Hyuck Im, Jeong Eon Lee, Seok Won Kim, Seok Jin Nam, Jonghan Yu, Yeon Hee Park

**Affiliations:** 1 Division of Hematology-Oncology, Department of Medicine, Samsung Medical Center, Sungkyunkwan University School of Medicine, Seoul, Korea; 2 Department of Health Sciences and Technology, SAIHST, Sungkyunkwan University School of Medicine, Seoul, Korea; 3 Samsung Genome Institute, Samsung Medical Center, Seoul, Korea; 4 Department of Surgery, Samsung Medical Center, Sungkyunkwan University School of Medicine, Seoul, Korea

**Keywords:** Cancer genomics, Translational research, Genetics research, Cancer genomics

## Abstract

Genomic analysis of structural variants(SVs) in breast cancer (BC) patients has been conducted, but the relationship between genomic alterations and BC prognosis remains unclear. We performed RNA sequencing of 297 early BC fresh-frozen tissues. We identified SVs using three tools (STAR.Arriba, STAR.fusion, and STAR.SEQR) with the COSMIC and Mitelman databases as guide references. We found a median of five to eight fusions per sample. In BC intrinsic subtypes, normal subtype had the fewest fusions (median: 1, interquartile range [IQR]: 0, 3) followed by luminal A (median: 5.5, IQR: 2.75, 10.25), luminal B (median: 9, IQR: 6, 16.5), HER2-enriched (median: 9, IQR: 6, 16.5) and basal (median 10, IQR: 6, 15.5) subtypes (*p* < 0.05). Intrachromosomal fusion was more frequent observed rather than interchromosomal fusion. In location, chromosome 17 had the most fusions followed by chromosome 1 and 11. When samples were divided into high and low fusion groups based on a cut-off value of 11 fusions, five-year event-free survival (5Y-EFS) was 68.1% in the high fusion group (*n* = 72) and 80.1% in the low fusion group (*n* = 125) (*p* = 0.024) while 75.6% among all patients (95% confidence interval: 0.699, 0.819). Among BC subtype, TNBCs with more fusions had shorter EFS compared to those with fewer fusions (5Y-EFS rate: 65.1% vs. 85.7%; *p* = 0.013) but no EFS differences were observed in other BC subtypes. ESTIMATE ImmuneScore was also associated with the number of fusions in TNBC (*p* < 0.005) and TNBCs with high ImmuneScore had better 5Y-EFS compared to those with low ImmuneScore (*p* = 0.041). In conclusion, diverse fusions were observed by BC subtype, and the number of fusions was associated with BC survival outcome and immune status in TNBC.

## Introduction

Breast cancer (BC) is the most frequently diagnosed malignancy in the world^1^. Recent advances in BC diagnosis and treatment modalities have enabled early diagnosis of BC and improved survival. Nevertheless, many BC patients have a dismal prognosis^2,3^.

Next-generation sequencing provides insight into the genetic history of cancers. For BC, the most frequently observed mutations in The Cancer Genome Atlas (TCGA) are *PIK3CA* and *TP53* somatic mutations; other genetic studies have described alterations of driver genes of BC including *MYC*, *CCND1*, *PTEN*, and *ERBB2*^4–6^.

These discoveries of genetic alterations in BC heralded the era of precision medicine and targeted therapy. Amplification of *ERBB2*, a traditional BC biomarker, has been treated with human epidermal growth factor receptor-2(HER-2)-targeted agents like trastuzumab, an anti-HER-2 monoclonal antibody^7–10^. In addition, a PIK3CA inhibitor, alpelisib, is now approved for treatment of patients with hormone receptor (HR)-positive metastatic BC harboring *PIK3CA* hotspot mutations^11–13^. Additionally, *ESR1* mutations, a resistance mechanism of aromatase inhibitors, can be repaired with a new-generation selective estrogen receptor degrader^14–16^.

Recent pan-cancer genomic data have revealed that BC was the most commonly structural variants (SVs) harboring cancer among various cancer types^17^. In addition, these SVs including fusion could be resistance mechanisms of therapy and also be therapeutic targets. For example, neurotrophin tyrosine receptor kinase (*NTRK)* gene fusions (*NTRK1*, *NTRK2*, or *NTRK3*) are oncogenic drivers in various tumor types^18^ that can be targeted by recently developed TRK inhibitors^19,20^ and *ESR1* fusions were resistant mechanism of endocrine therapy in HR-positive BC^21^. However, SVs, especially fusions in early BCs (EBCs) were rarely reported and the prognostic role of fusions was unrevealed.

In this study, we performed SV analysis using transcriptome sequencing of samples using 297 EBC samples. We evaluated fusions based on BC subtype and investigated the relationship between fusions and other genetic alterations. Lastly, we analyzed the prognostic value of fusions in EBC patients.

## Results

### Patients and tissue collection

**Fig. 1:**
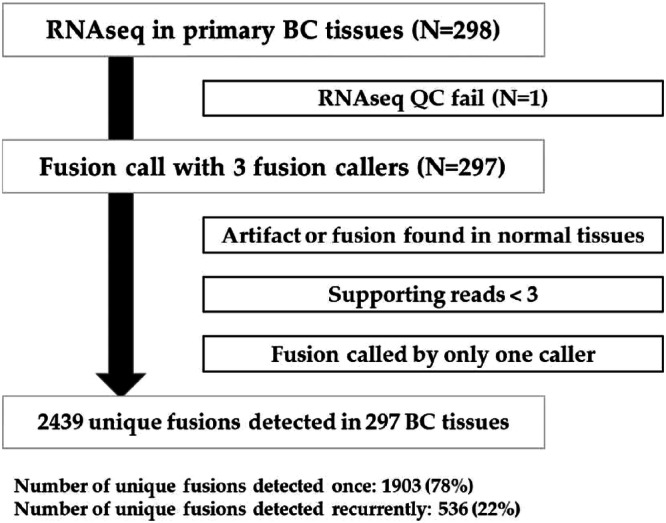
Consort diagram (*n* = 297).

We extracted RNA and performed RNAseq of tissue samples from 298 BCs enrolled in two translational studies (Fig. 1). Due to quality control failure in one sample, we evaluated RNAseq data from 297 early BCs.

Baseline characteristics of tissue samples are described in Table 1. In 297 BCs. median age at diagnosis of BC patients was 39.9 years (interquartile range [IQR]: 35.5–49.4); 81.1% of samples were collected in tissue biopsy in neoadjuvant (NAC) setting and 18.9% surgical specimens in an adjuvant setting. All BC specimens were harvested from the breast. With regard to BC subtype, 51.2% had triple-negative BC (TNBC), 22.6% HR + HER2-, 14.1% HR + HER2 + , and 12.1% HR-HER2 + . In NAC setting, 27.4% were HR + HER2-BC, 32.4% in HER2+ regardless of HR state and 40.2% in TNBC whereas 98.2% of young breast cancer(YBC) cohort were TNBC. We further evaluated the intrinsic subtype through PAM50 analyses: luminal A type was identified in 17.5% of samples, luminal B in 10.1%, basal-like in 49.5%, HER2 enriched in 18.5%, and normal in 4.4%.

**Table 1:** Sample characteristics (*N* = 297)

Characteristics	*N*(%)	Characteristics	*N*(%)
Age (median)	39.9	Subtype	
< 40 years old	150 (50.5)	HR + ^a^ HER2-^b^	67 (22.6%)
> 40 years old	147 (49.5)	HR + HER2+	42 (14.1%)
Patient cohort		HR-HER2+	36 (12.1%)
NAC^d^	241 (81.1)	TNBC^c^	152 (51.2%)
YBC^e^	56 (18.9)	Intrinsic subtype	
Organ status		Luminal A	52 (17.5%)
Breast	297 (100)	Luminal B	30 (10.1%)
Tissue status		Her2-enriched	55 (18.5%)
Biopsy	241 (81.1)	Basal-like	147 (49.5%)
Surgical specimen	56 (18.9)	Normal-like	13 (4.4%)
Treatment status^f^	(*n* = 197)		
Neoadjuvant CTx^g^	161 (81.7)	Subtype	
Adjuvant CTx.	52 (26.3)	HR + ^a^ HER2-^b^	38 (19.3)
Intrinsic subtype		HR + HER2+	26 (13.2)
Luminal A	25 (12.7)	HR-HER2+	20 (10.2)
Luminal B	22 (11.2)	TNBC^c^	113 (57.4)
Her2-enriched	33 (16.8)	Clinical stage	(*n* = 161)
Basal-like	112 (56.9)	Stage IIA	32 (19.9%)
Normal-like	5 (2.5)	Stage IIB	41 (25.5%)
Pathologic stage	(*n* = 36)	Stage IIIA	44 (27.3%)
Stage IA	17 (47.2%)	Stage IIIB	6 (3.7%)
Stage IIA	14 (28.9%)	Stage IIIC	31 (19.3%)
Stage IIB	5 (13.9%)	Unknown	7 (4.3%)

^a^hormone receptor; ^b^human epidermal growth factor rececptor-2; ^c^triple negative breast cancer; ^d^neoadjuvant cohort; ^e^young breast cancer cohort; ^f^the information of breast cancer treatment was restricted in 197 patients; ^g^Chemotherapy.

Among 297 BCs, we collected follow up survival data and treatment information in 197 BC patients (Table 1). In 197 BC patients, 161 patients were treated with NAC followed by curative surgery and 52 patients were treated with surgery followed by adjuvant treatment regarding BC subtypes. In 161 BCs in NAC cohort, clinical stage at diagnosis included 19.3% of clinical stage IIIC and 57.4% of patients were diagnosed as TNBC (Table 1). Details of BC subtypes and intrinsic subtypes were described in Supplementary Tables 2 and 3.

### Fusions according to BC characteristics

We analyzed the RNAseq of 297 tissue samples using three fusion detection software programs (Supplementary Table 4). First, we included fusions with more than three supporting reads (Fig. 1). We excluded fusions identified by only one program, artifacts, and fusions found in normal tissue.

We found a median of five to eight fusions (Supplementary Table 4). Among the three callers, STAR.Arriba detected the most fusions (median number of fusions: 8, IQR: 4–14), whereas STAR.Fusion (median: 7, IQR: 4–13) and STAR.SEQR (median: 5, IQR: 3–9) found the fewest. The median number of detected fusions per BC sample after filtering was 5 (IQR: 3–9) (Supplementary Table 5).

**Fig. 2:**
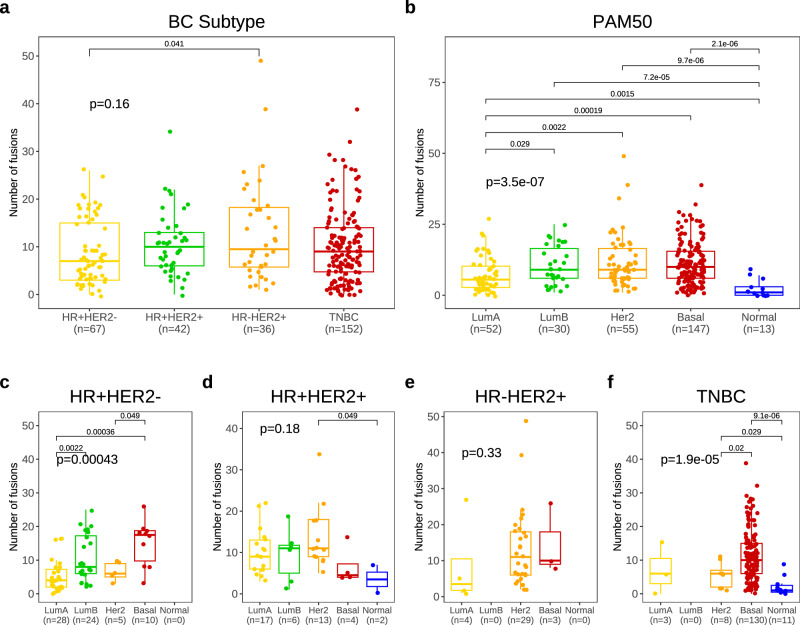
Number of fusions by breast cancer subtypes. Number of fusions according to (**a**). immunohistochemical breast cancer (BC) subtype and (**b**) PAM50 intrinsic subtype and (**c**–**f**) PAM50 intrinsic subtype in each breast cancer subtype (*n* = 297).

We also evaluated fusions according to BC subtype (Fig. 2a). HR + HER2- BC had the fewest fusions (median: 7, IQR: 3–15) compared to TNBC (median: 9, IQR: 4.75–14), HR-HER2 + BC (median: 9.5, IQR: 5.75–18.25), and HR + HER2 + BC (median: 10, IQR: 6–13) (*p* = 0.16). In intrinsic subtype, the normal-like subtype had the fewest fusions (median: 1, IQR: 0, 3) followed by the luminal A (median: 5.5, IQR: 2.75, 10.25), luminal B (median: 9, IQR: 6, 16.5), HER2-enriched (median: 9, IQR:6, 16.5) and basal-like (median 10, IQR: 6, 15.5) intrinsic subtypes, in ascending order (*p* < 0.05) (Fig. 2b).

Further intrinsic subtype analysis presented that basal-like subtype had more fusions compared to other intrinsic subtypes in HR + HER2- BC (median number of fusions of basal like subtype in HR + HER2-BC: 17.5, IQR:9.75–18.75) (Fig. 2c) and TNBC (median number:10, IQR: 6 -15) (Fig. 2f) (*p* < 0.05, respectively). However, in HER2 + BC regardless of HR status, there was no difference in number of fusions according to intrinsic subtype (ps > 0.05, respectively) (Fig. 2d, e).

**Fig. 3:**
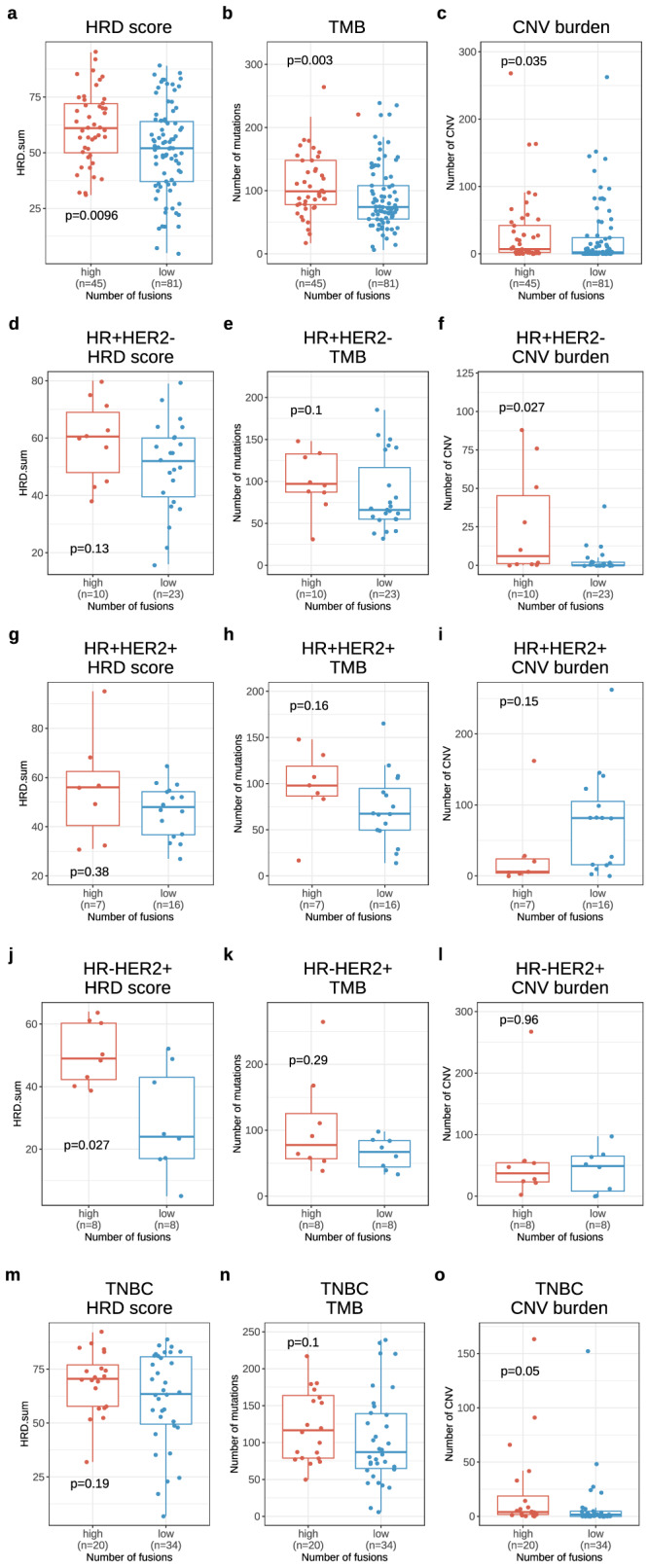
HRD, TMB and CNV burden according to number of fusions in breast cancer. **a** Homologous recombinant deficiency (HRD) score, **b** Tumoral mutational burden (TMB). **c** Copy number variant (CNV burden) between high (*n* = 45) and low (*n* = 81) number of fusions in all breast cancer (BC) (*n* = 126 which being performed both WTS and whole exome sequencing), **d** HRD score, **e** TMB. **f** CNV burden between high (*n* = 10) and low (*n* = 23) number of fusions in HR + HER2- BC, **g** HRD score. **h** TMB. **i** CNV burden between high (*n* = 7) and low (*n* = 16) number of fusions in HR + HER2 + BC. **j** HRD score. **k** TMB, **l** CNV burden between high (*n* = 8) and low (*n* = 8) number of fusions in HR-HER2 + BC. **m** HRD score. **n** TMB. **o** CNV burden between high (*n* = 20) and low (*n* = 34) number of fusions in TNBC.

Other genomic characteristics including homologous recombinant deficiency (HRD) score, tumor mutational burden(TNB) score, and copy number variant (CNV) were also evaluated for association with number of fusions using 126 BCs which being done whole exome sequencing (WES) analysis (Fig. 3a–c and supplementary Table 2 and 3). In this analysis, high HRD score, high TMB score and high CNV burden were positively correlated to number of fusions (*p* = 0.010, *p* = 0.003, and p = 0.035, respectively). In subspecific analyses according to BC subtype, CNV burden was associated with number of fusions in HR + HER2- subtype and TNBC whereas HRD was in HR-HER2+ subtype (Fig. 3d–o).

### Frequent fusions in early breast cancer

After filtering, we found 2439 unique fusions (Table 2). Among these events, there were 515 (21.1%) recurrent events, 365 (15.0%) known cancer-related fusions and 131 (5.4%) known BC-related fusions according to public databases including Mitelman and FusionAnnotator, which contained ChimerDB, COSMIC, and TCGA fusions^22,23^. With regard to chromosomes, chromosome 17 had most fusions followed by chromosome 1, 11, and 8 among 23 chromosomes. In addition, intrachromosomal fusion was detected more frequently than interchromosomal fusion (Table 3). The four chromosomes harboring the most fusions also harbored up to 70% of intrachromosomal fusions. *FBXL20*, *BCAS3*, *ERBB2*, and *IKZF3* were the most frequently detected fusion genes in chromosome 17. In total, 77 (3.2%) fusions were known recurrent fusions. The most commonly detected fusion event was FSIP1-AC013652.1 (Supplementary Table 6).

**Table 2:** Number of known and unknown fusions

	Unknown	Known	Sum
Private	1636 (67.1%)	288 (11.8%)	1924 (78.9%)
Recurrent	438 (18.0%)	77 (3.2%)	515 (21.1%)
Sum	2074 (85.0%)	365 (15.0%)	2439 (100.0%)

Number of fusion events (percentage of each fusions compared to the total number of fusion events).

**Table 3:** Number of inter/ intra-chromosomal fusions by chromosome

Chromosome	All fusions		Known fusions	
	Inter-chromosomal	Intra-chromosomal	Inter-chromosomal	Intra-chromosomal
chr1	135 (26.3%)	378 (73.7%)	1 (1.3%)	76 (98.7%)
chr2	85 (33.3%)	170 (66.7%)	1 (2.7%)	36 (97.3%)
chr3	86 (31.6%)	186 (68.4%)	0 (0%)	56 (100%)
chr4	65 (40.4%)	96 (59.6%)	0 (0%)	18 (100%)
chr5	90 (51.7%)	84 (48.3%)	0 (0%)	18 (100%)
chr6	94 (35.9%)	168 (64.1%)	0 (0%)	34 (100%)
chr7	77 (38.3%)	124 (61.7%)	0 (0%)	42 (100%)
chr8	100 (35.7%)	180 (64.3%)	0 (0%)	58 (100%)
chr9	67 (43.2%)	88 (56.8%)	0 (0%)	22 (100%)
chr10	77 (41.6%)	108 (58.4%)	1 (4.3%)	22 (95.7%)
chr11	70 (24.5%)	216 (75.5%)	0 (0%)	48 (100%)
chr12	86 (44.8%)	106 (55.2%)	0 (0%)	40 (100%)
chr13	40 (44.4%)	50 (55.6%)	0 (0%)	12 (100%)
chr14	34 (36.2%)	60 (63.8%)	0 (0%)	24 (100%)
chr15	56 (39.4%)	86 (60.6%)	0 (0%)	24 (100%)
chr16	58 (29.6%)	138 (70.4%)	0 (0%)	26 (100%)
chr17	154 (23.3%)	508 (76.7%)	1 (1.3%)	78 (98.7%)
chr18	32 (47.1%)	36 (52.9%)	1 (20%)	4 (80%)
chr19	63 (26.4%)	176 (73.6%)	1 (4%)	24 (96%)
chr20	70 (39.8%)	106 (60.2%)	0 (0%)	30 (100%)
chr21	38 (47.5%)	42 (52.5%)	0 (0%)	6 (100%)
chr22	42 (35.6%)	76 (64.4%)	0 (0%)	20 (100%)
chrX	43 (55.8%)	34 (44.2%)	0 (0%)	6 (100%)
chrY	0 (100%)	0 (0%)	0 (100%)	0 (0%)

Number of fusions located in each chromosome (percentage of inter/intra-chromosomal fusions located in each chromosome). Each fusion was counted twice based on chromosomal location of gene1 and gene2.

**Fig. 4:**
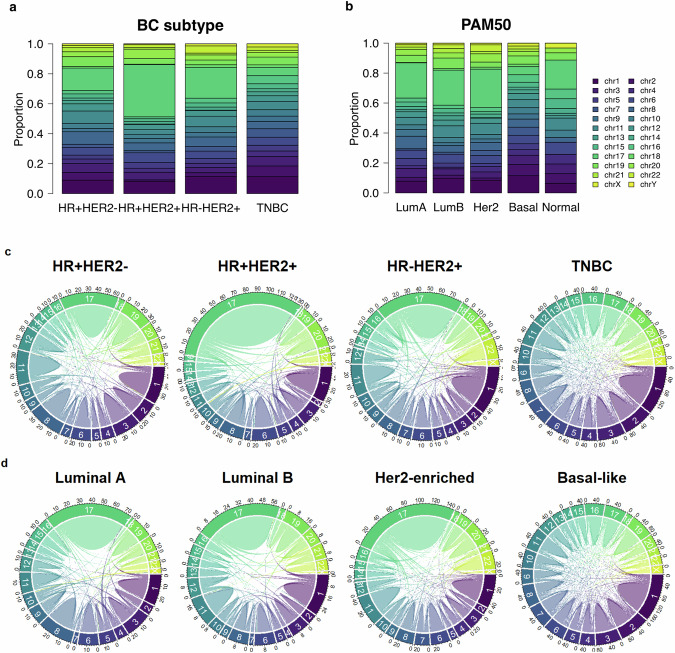
Proportion of fusion events in chromosomes. **a** The proportion of fusions in chromosomes according to HR + HER2- breast cancer (BC), HR + HER + BC, HR-HER2 + BC and triple negative breast cancer (TNBC), **b** The proportion of fusions in chromosomes according to luminal A, luminal B, HER2-enriched, basal and normal intrinsic subtype, **c** Circos plot for fusions according to HR + HER2- BC, HR + HER + BC, HR-HER2 + BC and TNBC, **d** Circos plot for fusions according to luminal A, luminal B, HER2-enriched, basal and normal intrinsic subtype.

We also analyzed fusions in chromosomes according to BC subtype and intrinsic subtype (Fig. 4). In BC subtype, the fusions in HR + HER2 + BC subtype mostly occurred in chromosome 17, while the fusions in TNBC were mostly observed in chromosome 1 but evenly distributed in whole chromosomes (*p* < 0.05) (Fig. 4a, c). In intrinsic subtypes, fusions in the basal-like subtypes mostly occurred in chromosome 1, while the other occurred in chromosome 17, respectively (*p* < 0.05) (Fig. 4b, d).

### Survival outcomes according to number of fusions

**Fig. 5:**
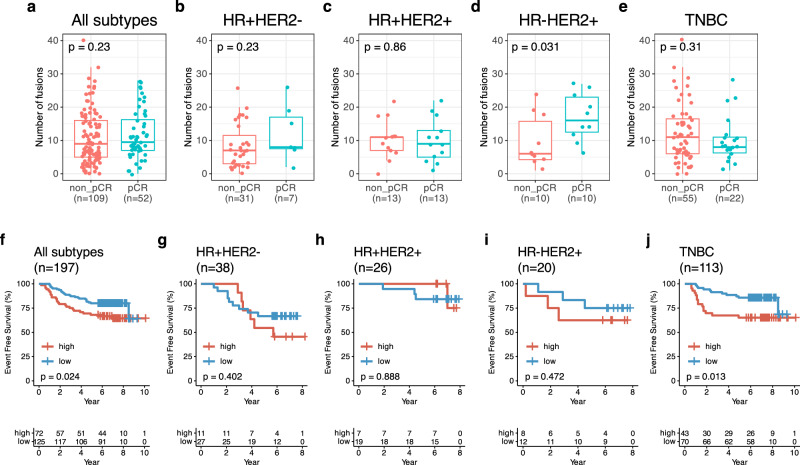
Treatment outcomes and number of fusion events. **a** Number of fusions between non-pathologic complete response(pCR) (*n* = 109) and pCR (*n* = 52) in all subtypes, **b** Number of fusions between non-pathologic complete response(pCR) (*n* = 31) and pCR (*n* = 7) in HR + HER2- BC. **c** Number of fusions between non-pathologic complete response(pCR) (*n* = 13) and pCR (*n* = 13) in HR + HER2 + BC. **d** Number of fusions between non-pathologic complete response(pCR) (*n* = 10) and pCR (*n* = 10) in HR-HER2 + BC and (**e**) number of fusions between non-pathologic complete response(pCR) (*n* = 55) and pCR (*n* = 22) in triple-negative breast cancer (TNBC). **f** Kaplan-Meier (KM) for event-free survival (EFS) according to high vs. low number of fusions (cut-off value: 0.6) in all subtypes (*n* = 197). **g** KM for EFS in HR + HER2- BC (*n* = 38), **h** KM for EFS in HR + HER2 + BC (*n* = 26). **i** KM for EFS in HR-HER2 + BC (*n* = 20) and (**j**) KM for EFS in TNBC (*n* = 113).

We further evaluated treatment outcomes according to fusions (Fig. 5). Only 197 BC patients having follow up survival data were enrolled in this analysis. For, survival analysis, we divided BCs into two groups according to number of fusions with a 0.6 cut-off value (11 fusions). The 0.6 cut off value was based on log-rank test with consecutive cutoff values in total BC patients and this was numerically 11 fusions (Supplementary Table 7).

Among these 197 patients, 143 patients in NAC cohort were evaluated for pathologic complete response(pCR) according to fusions (Fig. 5a–e). In this analysis, the number of fusions was not significantly different according to the pCR status with all patients (*p* = 0.23, Fig. 5a). In BC subtype, higher number of fusions was observed in pCR group compared to non-pCR group (*p* = 0.031) in HR-HER2 + BC subgroup (Fig. 5d) whereas HER2-enriched intrinsic subtype with pCR had lower fusions compared to those with non-pCR (*p* = 0.021) (Supplementary Fig. 1C). Moreover, luminal A subtype had higher fusions in those with pCR compared to those without pCR (*p* = 0.045) (Supplementary Fig. 1B). Otherwise, there was no significant difference of the number of fusions by pCR status in other BC subtypes and intrinsic subtypes (Fig. 5b–e and supplementary Fig. 1B, D)

Furthermore, we performed survival analysis in 197 EBC patients with 7 years of median follow up duration (Fig. 5f–j). The five-year event-free survival (5Y-EFS) rate was 75.6% in all patients (95% confidence interval [CI]: 0.699, 0.819) and the 5Y-EFS rate was 68.1% in the high fusion group (*n* = 72) and 80.0% in the low fusion group (*n* = 125) (*p* = 0.024) (Fig. 5f).

In survival analysis for fusions according to BC subtypes, TNBC with higher number of fusions (*n* = 43) had a 5Y-EFS of 65.1%, and that with low fusions, 85.7% (*n* = 70) (*p* = 0.013), while their 5Y-EFS was 77.9% (95% CI: 0.706, 0.859) (*n* = 113) (Fig. 5j). In non-TNBCs, they had a trend that high fusions were associated with poor EFS, but statistical significance was not observed (Fig. 5g–i).

Among five intrinsic subtypes, we analyzed EFS according to the fusions in four intrinsic subtypes because normal-like intrinsic subtype was only five. In the basal-like intrinsic subtype (*n* = 112), the five-year EFS rate was 78.6% (95% CI: 0.713, 0.866). The basal–high fusion group had five-year EFS of 64.6% (*n* = 40) versus 89.1% in the basal–low fusion group (*n* = 72) (*p* = 0.003) (Supplementary Fig. 1H) meanwhile there were no relationship between fusions and EFS among non-basal like intrinsic subtypes (Supplementary Fig. 1E–G)

**Fig. 6:**
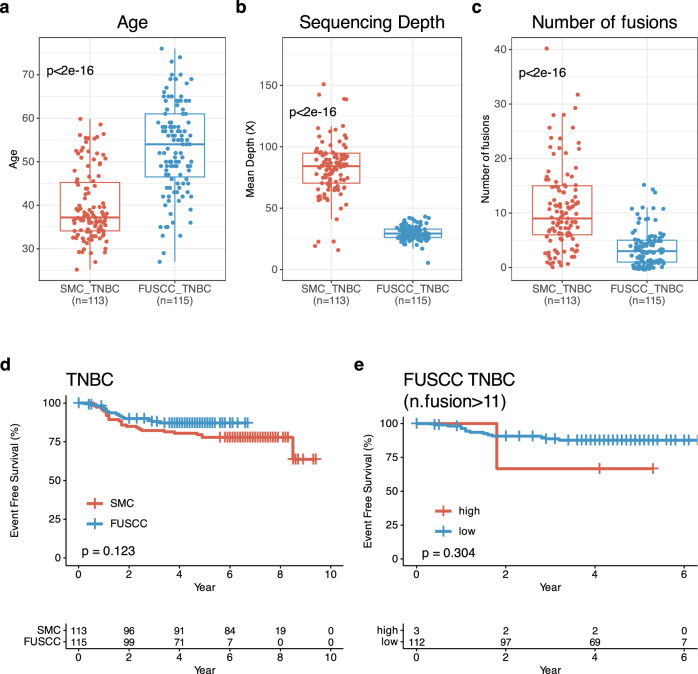
Valitation and comparision between our cohort and FUSCC cohort. Validation study with Fudan University Sanghai Cancer Center (FUSCC) TNBC. Comparison of (**a**). age between SMC TNBC at diagnosis (*n* = 113)_ and FUSCC TNBC at surgery (*n* = 115). **b** Whole transcriptome sequencing depth in SMC TNBC (*n* = 113) and FUSCC TNBC (*n* = 113). **c** Number of fusions depth in SMC TNBC (*n* = 113) and FUSCC TNBC (*n* = 113). **d** Kaplan-Meier(KM) of event-free survival(EFS) according to SMC (*n* = 113) and FUSCC TNBCs (*n* = 115). **e** KM for EFS of FUSCC TNBC according to high vs. low number of fusions (SMC cutoff value:0.6, *n* = 115).

For validating our data, we evaluated the association between fusions and 5Y-EFS rate in TNBC and basal-intrinsic subtypes using TNBC RNASeq data from Fudan University Sanghai Cancer Center (FUSCC). In total, we could use 115 TNBC RNASeq data from FUSCC TNBC cohort. In this validation cohort, median age of patients at BC diagnosis was 54.0 (IQR: 46.5, 61.0) and only twelve patients were under 40 years of age (*p* < 0.005) (Fig. 6a). In terms of mean depth of sequencing, FUSCC cohort had lower than our cohort (*p* < 0.005) and fewer fusions compared to our TNBC (median fusions: 3, IQR: 1, 5) (*p* < 0.005) (Fig. 6b, c). In terms of other clinical characteristics, we cannot find treatment setting regarding neoadjuvant and adjuvant settings.

There were similar EFS between FUSCC and our cohorts (Fig. 6d). Only three TNBCs had up to eleven fusions and therefore no significant EFS difference was observed (*p* = 0.304) even though three had lower 5Y-EFS rate compared to others in FUSCC cohort (Fig. 6e). Further analyses using different cut off values of fusions were performed in FUSCC cohort and the results showed consistently that more fusions in TNBCs was the surrogate marker of shorter EFS compared to those with fewer fusions (Supplementary Fig. 3).

### Immune status according to fusions

**Fig. 7:**
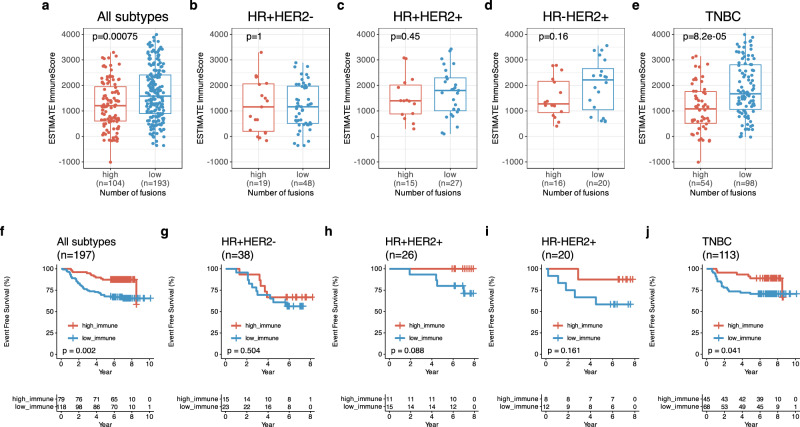
Survival outcome and ImmuneScore regarding number of fusion events. **a** ESTIMATE ImmuneScore between high (*n* = 104) and low (*n* = 193) fusion events in all subtypes. **b** ESTIMATE ImmuneScore between high (*n* = 19) and low (*n* = 48) fusion events in HR + HER2- BC, **c** ESTIMATE ImmuneScore between high (*n* = 15) and low (*n* = 27) fusion events in HR + HER2 + BC. **d** ESTIMATE ImmuneScore between high (*n* = 16) and low (*n* = 20) fusion events in HR-HER2 + BC and (**e**) ESTIMATE ImmuneScore between high (*n* = 54) and low (*n* = 98) fusion events in triple-negative breast cancer(TNBC), **f** Kaplan-Meier (KM) for event-free survival (EFS) according to ESTIMATE ImmuneScore in all subtypes. **g** KM for EFS in HR + HER2- BC, **h** KM for EFS in HR + HER2 + BC. **i** KM for EFS in HR-HER2 + BC and (**j**) KM for EFS in TNBC.

Afterwards, we performed the analysis for the association between ESTIMATE ImmuneScore and the number of fusions. In this analysis, high fusion group had a lower ImmuneScore than low fusion group with all patients (*p* < 0.001) (Fig. 7a). The TNBC–high fusion group had a lower ImmuneScore (median: 1079, IQR: 514, 1761) than the TNBC–low fusion group (median: 1673, IQR: 1057, 2809) (*p* < 0.001) (Fig. 7e) but non-TNBC subtype did not have a relationship (Fig. 7b–d). Likewise, basal-like intrinsic subtype had a relationship between ImmuneScore and number of fusions (p = 0.0016, Supplementary Fig. 2D) but there was no relationship in non-basal intrinsic subtypes (Supplementary Fig. 2A–C). In survival analysis for ImmuneScore, high ImmuneScore group had better EFS than low ImmuneScore group (*p* = 0.002, Fig. 7f). TNBC patients with a high ImmuneScore had better EFS compared to that with a low ImmuneScore (five-year EFS of TNBC with high vs. low ImmuneScore: 91.9% [95% CI: 0.835, 1.00] vs. 71.1% [95% CI: 0.616, 0.820]) (Fig. 7j). This trend was also observed in basal-like intrinsic subtypes (p = 0.019, Supplementary Fig. 2H). Withal, ImmuneScore did not affected EFS in non-TNBCs (Fig. 7g–i) as well as non-basal intrinsic subtypes (Supplementary Fig. 2E–G).

## Discussion

In this study, we searched for fusions in tissue samples from 297 EBCs and revealed that higher fusions were associated with shorter EFS in EBC, especially TNBC or basal-like intrinsic subtypes. The median number of fusions was nine and the incidence of fusions varied not only by BC subtype but also by intrinsic subtype. Among these fusions, 40 events were included in both the Mitelman and COSMIC fusion databases. Based on the Mitelman database, 208 of the detected fusions have previously been found in various cancers and 90 specifically in BC. Among these 90 fusions, 58 were found in TNBC, 15 in ER + HER2-, 12 in ER + HER2 + , and 11 in ER-HER2 + BC. By intrinsic subtype, there were 60 fusions in basal-like, 12 in luminal A, 8 in luminal B, and 18 in HER2-enriched subtypes.

Gene rearrangement is not frequently reported in BC, especially EBC^24,25^. Previous studies on fusions in BC suggested that the HR-HER2 + BC and TNBC subtypes showed more frequent fusions compared with the HR + HER2- and HR + HER2+ subtypes. They also reported only a small number of fusions in BC compared to other types of cancer. In our study, HR + HER2- BC had slightly fewer fusions compared to other BC subtypes. In intrinsic subtype, the basal-like subtype had most fusions followed by the HER2-enriched and luminal B subtypes, whereas luminal A and normal-like subtypes had few fusions.

In addition to the number of fusions, the loci of fusions also depended on intrinsic subtype. In basal-like BC, fusions most commonly occurred in chromosome 1 but we similarly observed fusions in whole chromosomes, whereas other intrinsic subtypes harbored fusions mostly in chromosome 17. A recent study revealed that translocations were caused by oncogene amplification as an early genetic structural alteration event. Specifically, *ERBB2* amplification also suggested that *ERBB2* translocation was an interchromosomal event^26^. Our research also indicated that *ERBB2* amplification was related to fusions in chromosome 17. However, intrachromosomal events were more frequently observed in our study compared to interchromosomal events. In the TCGA cohort, 1.4% of *ERBB2* fusions occurred not in HR-HER2 + BC, but in other subtypes^27,28^. *ERBB2* fusion is observed in non-small-cell lung cancer (NSCLC)^29^, accounting for 0.3% of *ERBB2* fusions. The pan-HER tyrosine kinase inhibitor afatinib has been used to effectively treat NSCLC harboring *ERBB2* fusions.

*ESR1* fusions were observed in two HR + HER2- BCs and one HR + HER2 + BC. By intrinsic subtype, two were in luminal B and one was in HER2. All were intrachromosomal events and two of three fusions were occurred in ligand binding domain of *ESR1*. In the TCGA PanCancer Atlas Project, fusions affecting *ESR1* were infrequent in BC (0.8%), and counterpart genes varied^27^. Recent study also suggested most recurrent luminal B subtype enriched fusions were including *ESR1* in metastatic setting and 5% of HR positive treatment refractory metastatic BC (MBC) harbored *ESR1* fusion^30^. Previous functional study of *ESR1* fusion in HR + MBC suggested that the fusion transcriptome triggered endocrine resistance and promoted metastasis^21^. Especially, two of three patients with *ESR1* fusion had experienced BC recurrence within five years and therefore, *ESR1* fusion may have a role of primary resistance mechanism for adjuvant endocrine therapy in HR positive EBC.

We also found two *NTRK* fusions, CCL28-NTRK1 and NTRK2-BANCR in TNBC. Recent studies of *NTRK* fusion focused on treatment response to new TRK inhibitors in tumors harboring *NTRK* fusion^31,32^. These drugs were effective for treating all types of cancer harboring *NTRK* fusions. Although *NTRK* fusion is a rare genetic alteration in BC, TRK inhibitors would likely be similarly effective in such cases.

We found that fusions were associated with EFS in EBC patients. Specifically, there was a significant association between number of fusions and five-year EFS in TNBC and basal-like subtype. Previous genomic studies suggested that tumoral mutation burden was associated with survival outcome, but this also depended on nodal stage^33^. Previous studies as well as this study have suggested that immune signature is related to survival outcomes of TNBC, but this signature was calculated based on RNA expression data and difficult to reproduce in other TNBC cohorts^33,34^. Fusions in TNBC were not associated with HRD, TMB burden in our study and therefore fusion itself was associated with EFS in TNBC. In RNASeq data from FUSCC cohort, our finding was also present in validation cohort even though they had fewer fusions in their cohort. The difference of number of fusions between two cohort would depend on depth of sequencing. In spite of this difference characteristics between two cohort, the trend for more fusions shorter EFS was consecutively similar.

Lastly, ImmuneScore was negatively correlated to the number of fusions in TNBC and basal-like intrinsic subtypes. In our study, CNV burden was also related to the number of fusions and this suggested that genomic alterations including CNV burden and SVs might be associated to tumor microenvironment.

Our cohort included high stage EBCs which needed neoadjuvant chemotherapy and young patients who had worse prognosis rather than older patients^6^. In terms of BC subtype, up to 50% of TNBCs were in this study. Therefore, our cohort had relatively worse survival outcome compared to that of other EBC cohort, although all patients had been received cytotoxic chemotherapy in neoadjuvant or adjuvant setting. In addition, false positive fusion calls may exist even though we used three software for calling fusions and then strictly cut off fusions. Nevertheless, our study suggested that the genomic structural characteristics in EBC with unfavorable survival outcome. In conclusion, we investigated structural variants in tumors from EBC patients. Consistent with previous studies, median number of fusions was lower than ten but TNBC or basal-like intrinsic subtype harbored more fusions rather than other subtypes. In addition, fusions occurred across various chromosomes in TNBC, and survival outcomes were associated with the number of fusions. Further functional validation is warranted to confirm the role of these fusions.

## Methods

### Tissue collection

We collected BC tissue samples from patients who participated in explorative trials at Samsung Medical Center from December 2013 to June 2020. The institutional review board of Samsung Medical Center approved the study protocol (IRB No: 2022-05-004) and participants provided written informed consent to take part in study (Supplementary Table 1). This study was performed in accordance with the Declaration of Helsinki^6,35^. In NAC cohort, patients had received NAC (four cycles of adriamycin and cyclophosphamide followed by four cycles of docetaxel) and trastuzumab and/or pertuzumab for HER2 + BC. In YBC cohort, 16 patients had received NAC and 38 patients had undergone curative surgery followed by adjuvant chemotherapy per protocol. Radiotherapy, endocrine therapy and targeted therapy for HER2 + BC had also performed per standard therapeutic guideline.

All available hematoxylin and eosin-stained slides for fresh-frozen tissues were collected. All pathology specimens were reviewed by independent pathologists to determine tumor histology and immunohistochemical (IHC) findings (estrogen receptor [ER] and progesterone receptor [PgR] expression and HER2 overexpression). ER and PgR positivity, the presence of either of which was defined as HR positivity, was defined by Allred scores ranging from 3–8 based on IHC using antibodies to ER (Immunotech, Marseille, France) and PgR (Novocastra Laboratories Ltd., Newcastle upon Tyne, UK), respectively. HER2 status was evaluated using a specific antibody (Dako, Glostrop, Denmark), fluorescent in situ hybridization (FISH), or silver in situ hybridization (SISH). Grade 0/1 HER2 on IHC was defined as a negative result, and grade 3 was defined as a positive result. Amplification of HER2 was confirmed by FISH or SISH if HER2 was rated as grade 2 on IHC. TNBC was defined as a negative result for ER/PgR and HER2.

### Whole transcriptome sequencing (WTS)

Total RNA from fresh-frozen tumor tissues was extracted with an RNeasy Mini Kit (Qiagen, Hilden, Germany). Nucleic acid extraction was performed according to the manufacturer’s instructions. The quality and quantity of extracted nucleic acids were evaluated using Nanodrop 8000 UV–Vis spectrometer (Thermo Fisher Scientific, Waltham, MA, USAQubit® 3.0 Fluorometer (Life Technologies, Inc., Carlsbad, CA, USA), and 4200 TapeStation (Agilent Technologies, Inc., Santa Clara, CA, USA). Sequencing libraries were prepared with TruSeq RNA Sample Preparation Kit v2 from fresh-frozen tissues (Illumina, Inc., San Diego, CA, USA), following the manufacturer’s protocols. Paired-end sequencing of the RNA libraries was performed on a HiSeq 2500 Sequencing Platform (Illumina, Inc.).

### Fusion detection, ImmuneScore and PAM50 subtyping

Fusion was predicted from RNAseq employing three fusion detection software programs with default parameters: STAR.Arriba v2.0.0^36^, STAR.fusion v1.9.1 (https://github.com/STAR-Fusion), and STAR.SEQR v0.6.7^37^. Reads aligned using STAR-2.7.6a served as input for fusion callers. Sequencing reads were aligned to the human reference sequence hg38. Fusions flagged as red herrings were filtered out based on healthy tissue samples or gene homology databases (GTEx_recurrent_StarF2019, BodyMap, DGD_PARALOGS, Greger_Normal, and Babiceanu_Normal). Read-through fusions, considered artifacts, were excluded. To eliminate false-positive fusions, we removed those with fewer than three supporting reads or without any split reads^38^. Fusion calls predicted by two or more of the three fusion detection programs were further analyzed. Fusions were annotated as known fusions, as reported in public databases including Mitelman 2023^22^, ChimerDB, COSMIC and TCGA fusions in the FusionAnnotator fusion_lib.Mar2021.dat^39^ leveraging the CTAT Human Fusion Lib database release v0.3.0. Known fusions in BC were manually reviewed on binary alignment and mapping (BAM) files using Integrative Genomic Viewers (https://software.broadinstitute.org/software/igv/)^40^. Fusions were visualized as the Circos plot using circlize in the R package (version 0.4.14). ImmuneScore was calculated using ESTIMATE R package (version 1.0.13) to estimate the immune microenvironment scores for each samples with gene expression values TPM (transcript per million). Patients with ImmuneScore higher than 60% of the total 297 samples were grouped as having a high ImmuneScore^41^. PAM50 intrinsic subtype was performed using Genefu R package (v2.26.1) with the gene expression data^42^.

### Whole exome sequencing (WES)

Pathologists determined tumor purity by reviewing tumor specimens, and samples with low tumor purity (cut-off, 20%) were excluded from sequencing. Genomic DNA was extracted from fresh-frozen tissues using the QIAamp DNA mini kit (Qiagen). Genomic DNA from peripheral blood was extracted using the QIAamp DNA blood maxi kit (Qiagen). Total RNA from fresh-frozen tumor tissues was extracted with an RNeasy mini kit (Qiagen) according to the manufacturer’s instructions. The quality and quantity of extracted nucleic acids were evaluated using the NanoDrop™ 8000 UV–Vis spectrometer (Thermo Fisher Scientific, Waltham, MA, USA), Qubit® 3.0 fluorometer (Thermo Fisher Scientific), and 4200 TapeStation (Agilent Technologies, Inc.).

High-quality gDNA in matched tumor and blood samples was sheared with an S220 ultra-sonicator (Covaris, Inc., Woburn, MA, USA) and used to construct a library with the SureSelect XT Human All Exon v5 and SureSelect XT reagent kit, HSQ (Agilent Technologies, Inc.), according to the manufacturer’s protocol. Libraries were pooled, denatured, and sequenced in 100-bp paired-end mode using the HiSeq rapid SBS kit v2 (200 Cycles) and HiSeq rapid PE cluster kit v2 on the Illumina HiSeq 2500 platform (Illumina, Inc.).

Reads were aligned to the human reference genome (hg19) using the Burrows–Wheeler alignment tool (BWA) v0.7.17^43^. Sequence alignment and mapping (SAM) files were converted into BAM files using SAMtools v1.6. Duplicate reads were removed using Picard v2.9.4, base quality was recalibrated, and local realignment was optimized using the Genome Analysis toolkit (GATK) v4.0.2.1^44^. SNVs and indels were identified using MuTect2 v4.0.2.1. Copy number alteration was estimated by CONTRA v2.0.4^45,46^.

### Tumor mutation burden and homologous recombination deficiency

The TMB (mutation load) was defined as the sum of the number of non-synonymous SNVs and indels. Genomic scar scores, including telomeric allelic imbalance (Telomeric.AI), loss of heterozygosity (HRD-LOH), and the number of large-scale transitions (LST), were determined using the scarHRD R package v0.1.0^47^. The sum of these three scores was referred to as the HRD score and indicated HRD status.

### Validation study

To validate our finding of the association between fusions and EFS in TNBC and basal-intrinsic subtypes, we used TNBC whole transcriptome sequencing data from Fudan University Sanghai Cancer Center (FUSCC)^48^. In total, we could use 115 TNBC RNASeq data from BC cohort (SRA accession number: SRP157974) for fusion detection applying the same softwares and filtering steps for SMC data described above. Recurrence-free survival was estimated with the number of fusions by log-rank test.

### Statistical analyses

For survival analysis, EFS was estimated using the Kaplan–Meier method by log-rank test with survminer in the R package (version 0.4.9). EFS was defined as the day between BC diagnosis and the first recurrence events including local and distant metastases, contralateral BC development, and BC specific death. High vs. low cutoff values were investigated by estimating survival differences with log-rank test using consecutive cutoff changes from 0.1 to 0.9 for all patients and various subgroups, and selected which covered the most subgroups (Supplementary Table 9). Patients with a fusion count exceeding 60% of those of patients analyzed were classified as having a high fusion burden. Patients exhibiting an ImmuneScore higher than 60% of those of patients analyzed were categorized as having a high ImmuneScore. The five-year EFS rate was calculated, including a 95% confidence interval (CI). All statistical analyses were conducted using R version 4.1.2. Adjusted p-values, calculated using the false discovery rate, were employed to determine statistical significance. P-values less than 0.05 from Wilcoxon rank sun test, Kruskal-Wallis tests or correlation tests were deemed statistically significant.

### Supplementary information

Supplementary Materials

Supplementary Table 8-9

## Data Availability

Sequence data of transcriptome sequencing have been deposited in the EGA with controlled access: the accession number EGAD00001004487 of NAC study and EGAD00001003776 of YBC study.

## Code availability

Fusion was predicted using STAR.Arriba (v2.0.0), STAR.fusion (v1.9.1), and STAR.SEQR (v0.6.7) with default parameters. SNVs and indels were identified using MuTect2 (v4.0.2.1), and copy number alteration was estimated by CONTRA (v2.0.4) with default parameters. All analyses were carried out using the circlize (v0.4.14), ESTIMATE (v1.0.13), genefu (v2.26.1), scarHRD (v0.1.0), survminer (v0.4.9) packages for the R software (v4.1.2). The R scripts used to analyse the data and draw the figures are available from the corresponding author on request.

## References

[1] Sung, H. et al. Global Cancer Statistics 2020: GLOBOCAN Estimates of Incidence and Mortality Worldwide for 36 Cancers in 185 Countries. *CA Cancer J. Clin.* **71**, 209–249 (2021). 33538338 10.3322/caac.21660

[2] Society, A. C. *Cancer Facts & Figures 2021* (2021).

[3] Ruhl J. L. et al. (eds.) Summary Stage 2018: Codes and Coding Instructions, National Cancer Institute, Bethesda, MD, 2018. (2018).

[4] Cancer Genome Atlas, N. Comprehensive molecular portraits of human breast tumours. *Nature* **490**, 61–70 (2012). 23000897 10.1038/nature11412 PMC3465532

[5] Nik-Zainal, S. et al. Landscape of somatic mutations in 560 breast cancer whole-genome sequences. *Nature* **534**, 47–54 (2016). 27135926 10.1038/nature17676 PMC4910866

[6] Kan, Z. et al. Multi-omics profiling of younger Asian breast cancers reveals distinctive molecular signatures. *Nat. Commun.* **9**, 1725 (2018). 29713003 10.1038/s41467-018-04129-4 PMC5928087

[7] Slamon, D. J. et al. Use of chemotherapy plus a monoclonal antibody against HER2 for metastatic breast cancer that overexpresses HER2. *N. Engl. J. Med.* **344**, 783–792 (2001). 11248153 10.1056/NEJM200103153441101

[8] Baselga, J. et al. Pertuzumab plus trastuzumab plus docetaxel for metastatic breast cancer. *N. Engl. J. Med.* **366**, 109–119 (2012). 22149875 10.1056/NEJMoa1113216 PMC5705202

[9] Verma, S. et al. Trastuzumab emtansine for HER2-positive advanced breast cancer. *N. Engl. J. Med.* **367**, 1783–1791 (2012). 23020162 10.1056/NEJMoa1209124 PMC5125250

[10] Modi, S. et al. Trastuzumab Deruxtecan in Previously Treated HER2-Positive Breast Cancer. *N. Engl. J. Med.* **382**, 610–621 (2020). 31825192 10.1056/NEJMoa1914510 PMC7458671

[11] Andre, F. et al. Alpelisib for PIK3CA-Mutated, Hormone Receptor-Positive Advanced Breast Cancer. *N. Engl. J. Med.* **380**, 1929–1940 (2019). 31091374 10.1056/NEJMoa1813904

[12] Andre, F. et al. Alpelisib plus fulvestrant for PIK3CA-mutated, hormone receptor-positive, human epidermal growth factor receptor-2-negative advanced breast cancer: final overall survival results from SOLAR-1. *Ann. Oncol.* **32**, 208–217 (2021). 33246021 10.1016/j.annonc.2020.11.011

[13] Narayan, P. et al. FDA Approval Summary: Alpelisib Plus Fulvestrant for Patients with HR-positive, HER2-negative, PIK3CA-mutated, Advanced or Metastatic Breast Cancer. *Clin. Cancer Res.* **27**, 1842–1849 (2021). 33168657 10.1158/1078-0432.CCR-20-3652 PMC8535764

[14] Razavi, P. et al. The Genomic Landscape of Endocrine-Resistant Advanced Breast Cancers. *Cancer Cell* **34**, 427–438 e426 (2018). 30205045 10.1016/j.ccell.2018.08.008 PMC6327853

[15] Shomali, M. et al. SAR439859, a Novel Selective Estrogen Receptor Degrader (SERD), Demonstrates Effective and Broad Antitumor Activity in Wild-Type and Mutant ER-Positive Breast Cancer Models. *Mol. Cancer Ther.* **20**, 250–262 (2021). 33310762 10.1158/1535-7163.MCT-20-0390

[16] Shao, P. A New Era in ER+ Breast Cancer: Best-in-Class Oral Selective Estrogen Receptor Degrader (SERD) Designed as an Endocrine Backbone Treatment. *J. Med Chem.* **64**, 11837–11840 (2021). 34339201 10.1021/acs.jmedchem.1c01268

[17] Li, Y. et al. Patterns of somatic structural variation in human cancer genomes. *Nature* **578**, 112–121 (2020). 32025012 10.1038/s41586-019-1913-9 PMC7025897

[18] Yoshino, T. et al. JSCO-ESMO-ASCO-JSMO-TOS: international expert consensus recommendations for tumour-agnostic treatments in patients with solid tumours with microsatellite instability or NTRK fusions. *Ann. Oncol.* **31**, 861–872 (2020). 32272210 10.1016/j.annonc.2020.03.299

[19] Hemming, M. L. et al. Response and mechanisms of resistance to larotrectinib and selitrectinib in metastatic undifferentiated sarcoma harboring oncogenic fusion of NTRK1. *JCO Precis Oncol.* **4**, 79–90 (2020). 32133433 10.1200/PO.19.00287 PMC7055910

[20] Murray, B. W. et al. Molecular Characteristics of Repotrectinib That Enable Potent Inhibition of TRK Fusion Proteins and Resistant Mutations. *Mol. Cancer Ther.* **20**, 2446–2456 (2021). 34625502 10.1158/1535-7163.MCT-21-0632 PMC9762329

[21] Lei, J. T. et al. Functional Annotation of ESR1 Gene Fusions in Estrogen Receptor-Positive Breast Cancer. *Cell Rep.* **24**, 1434–1444 e1437 (2018). 30089255 10.1016/j.celrep.2018.07.009 PMC6171747

[22] Mitelman, F., Johansson, B. & Mertens, F. “Mitelman Database of Chromosome Aberrations and Gene Fusions in Cancer” from https://mitelmandatabase.isb-cgc.org (2023).

[23] Jang, Y. E. et al. ChimerDB 4.0: an updated and expanded database of fusion genes. *Nucleic Acids Res.* **48**, D817–D824 (2020). 31680157 10.1093/nar/gkz1013 PMC7145594

[24] Fimereli, D. et al. Genomic hotspots but few recurrent fusion genes in breast cancer. *Genes Chromosomes Cancer* **57**, 331–338 (2018). 29436103 10.1002/gcc.22533

[25] Veeraraghavan, J., Ma, J., Hu, Y. & Wang, X. S. Recurrent and pathological gene fusions in breast cancer: current advances in genomic discovery and clinical implications. *Breast Cancer Res. Treat.* **158**, 219–232 (2016). 27372070 10.1007/s10549-016-3876-y PMC4979600

[26] Lee, J. J. et al. ERalpha-associated translocations underlie oncogene amplifications in breast cancer. *Nature* **618**, 1024–1032 (2023). 37198482 10.1038/s41586-023-06057-w PMC10307628

[27] Gao, Q. et al. Driver Fusions and Their Implications in the Development and Treatment of Human Cancers. *Cell Rep.* **23**, 227–238.e223 (2018). 29617662 10.1016/j.celrep.2018.03.050 PMC5916809

[28] Consortium, A. P. G. AACR Project GENIE: Powering Precision Medicine through an International Consortium. *Cancer Discov.* **7**, 818–831 (2017). 28572459 10.1158/2159-8290.CD-17-0151 PMC5611790

[29] Chunwei Xu. et al. Real-world large-scale study of ERBB2 gene fusions and its response to afatinib in Chinese non-small cell lung cancer (NSCLC): A multicenter study. *J Clin Oncol.* 37, e13002 (2019).

[30] Nolan Priedigkeit. GS03-09 Characterization and proposed therapeutic exploitation of fusion RNAs in metastatic breast cancers. San Antonio Breast Cancer Symposium (2023).

[31] Drilon, A. et al. Efficacy of Larotrectinib in TRK Fusion-Positive Cancers in Adults and Children. *N. Engl. J. Med.* **378**, 731–739 (2018). 29466156 10.1056/NEJMoa1714448 PMC5857389

[32] Demetri, G. D. et al. Updated Integrated Analysis of the Efficacy and Safety of Entrectinib in Patients with NTRK Fusion-Positive Solid Tumors. *Clin Cancer Res.* **28**, 1302–1312 (2022). 35144967 10.1158/1078-0432.CCR-21-3597 PMC9365368

[33] Ascierto, M. L. et al. A signature of immune function genes associated with recurrence-free survival in breast cancer patients. *Breast Cancer Res. Treat.* **131**, 871–880 (2012). 21479927 10.1007/s10549-011-1470-x PMC3431022

[34] Kim, J. Y. et al. Prognostication of a 13-immune-related-gene signature in patients with early triple-negative breast cancer. *Breast Cancer Res. Treat.* **184**, 325–334 (2020). 32812178 10.1007/s10549-020-05874-1

[35] Park, Y. H. et al. Chemotherapy induces dynamic immune responses in breast cancers that impact treatment outcome. *Nat. Commun.* **11**, 6175 (2020). 33268821 10.1038/s41467-020-19933-0 PMC7710739

[36] Uhrig, S. et al. Accurate and efficient detection of gene fusions from RNA sequencing data. *Genome Res.* **31**, 448–460 (2021). 33441414 10.1101/gr.257246.119 PMC7919457

[37] Weigman, S. et al. STAR-SEQR: Accurate fusion detection and support for fusion neoantigen applications. *Cancer Res.* **78**, 2296 (2018). 10.1158/1538-7445.AM2018-2296

[38] Haas, B. J. et al. Accuracy assessment of fusion transcript detection via read-mapping and de novo fusion transcript assembly-based methods. *Genome Biol.* **20**, 213 (2019). 31639029 10.1186/s13059-019-1842-9 PMC6802306

[39] Dehghannasiri, R. et al. Improved detection of gene fusions by applying statistical methods reveals oncogenic RNA cancer drivers. *Proc. Natl Acad. Sci. USA* **116**, 15524–15533 (2019). 31308241 10.1073/pnas.1900391116 PMC6681709

[40] Robinson, J. T. et al. Integrative genomics viewer. *Nat. Biotechnol.* **29**, 24–26 (2011). 21221095 10.1038/nbt.1754 PMC3346182

[41] Yoshihara, K. et al. Inferring tumour purity and stromal and immune cell admixture from expression data. *Nat. Commun.* **4**, 2612 (2013). 24113773 10.1038/ncomms3612 PMC3826632

[42] Gendoo, D. M. et al. Genefu: an R/Bioconductor package for computation of gene expression-based signatures in breast cancer. *Bioinformatics* **32**, 1097–1099 (2016). 26607490 10.1093/bioinformatics/btv693 PMC6410906

[43] Li, H. & Durbin, R. Fast and accurate short read alignment with Burrows-Wheeler transform. *Bioinformatics* **25**, 1754–1760 (2009). 19451168 10.1093/bioinformatics/btp324 PMC2705234

[44] DePristo, M. A. et al. A framework for variation discovery and genotyping using next-generation DNA sequencing data. *Nat. Genet* **43**, 491–498 (2011). 21478889 10.1038/ng.806 PMC3083463

[45] Mermel, C. H. et al. GISTIC2.0 facilitates sensitive and confident localization of the targets of focal somatic copy-number alteration in human cancers. *Genome Biol.* **12**, R41 (2011). 21527027 10.1186/gb-2011-12-4-r41 PMC3218867

[46] Li, J. et al. CONTRA: copy number analysis for targeted resequencing. *Bioinformatics* **28**, 1307–1313 (2012). 22474122 10.1093/bioinformatics/bts146 PMC3348560

[47] Sztupinszki, Z. et al. Migrating the SNP array-based homologous recombination deficiency measures to next generation sequencing data of breast cancer. *NPJ Breast Cancer* **4**, 16 (2018). 29978035 10.1038/s41523-018-0066-6 PMC6028448

[48] Jiang, Y. Z. et al. Genomic and Transcriptomic Landscape of Triple-Negative Breast Cancers: Subtypes and Treatment Strategies. *Cancer Cell* **35**, 428–440.e425 (2019). 30853353 10.1016/j.ccell.2019.02.001

