# Supplementary material for: Prognostic value of structural variants in early breast cancer patients
Source: NPJ Breast Cancer. 2024 Jul 27;10:64. doi: 10.1038/s41523-024-00669-9 (PMC11283467; doi:10.1038/s41523-024-00669-9)
Supplement: Supplementary file 1 — Supplementary Materials [file 41523_2024_669_MOESM1_ESM.pdf]

Supplementary Fig. 1: Number of fusions according to pathologic complete response after neoadjuvant chemotherapy in **a** All subtypes (n=161), **b** Luminal A (n=25), **c** Luminal B (n=22), **d** Her2-enriched (n=31) and **e** Basal-like (n=78) intrinsic subtype BCs. Kaplan-Meier for event-free survival according to high vs. low number of fusions in **f** All subtypes (n=197), **g** Luminal A (n=25), **h** Luminal B (n=25), **i** Her2-enriched (n=33) and **j** Basal-like (n=112) intrinsic subtype BCs. Normal-like BCs (n=5) not shown.

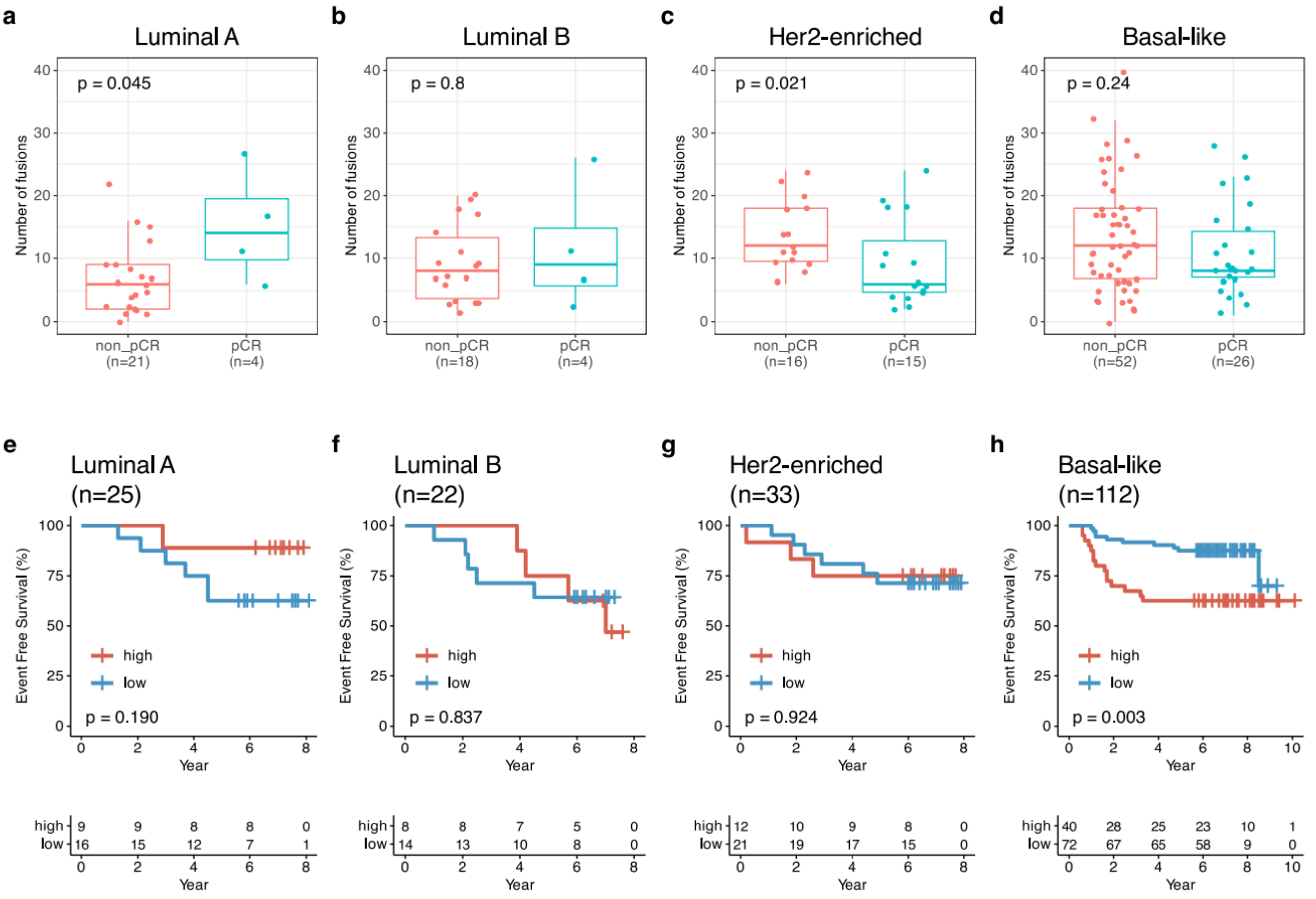

Supplementary Fig. 2: ESTIMATE ImmuneScore according to fusions in **a** All subtypes (n=297), **b** Luminal A (n=52), **c** Luminal B (n=30), **d** Her2-enriched (n=55) and **e** Basal-like (n=147) intrinsic subtype BCs. Kaplan-Meier for event-free survival according to high vs. low ImmuneScore in **f** All subtypes (n=197), **g** Luminal A (n=25), **h** Luminal B (n=25), **i** Her2-enriched (n=33) and **j** Basal-like (n=112) intrinsic subtype BCs. Normal-like BCs (n=5) not shown.

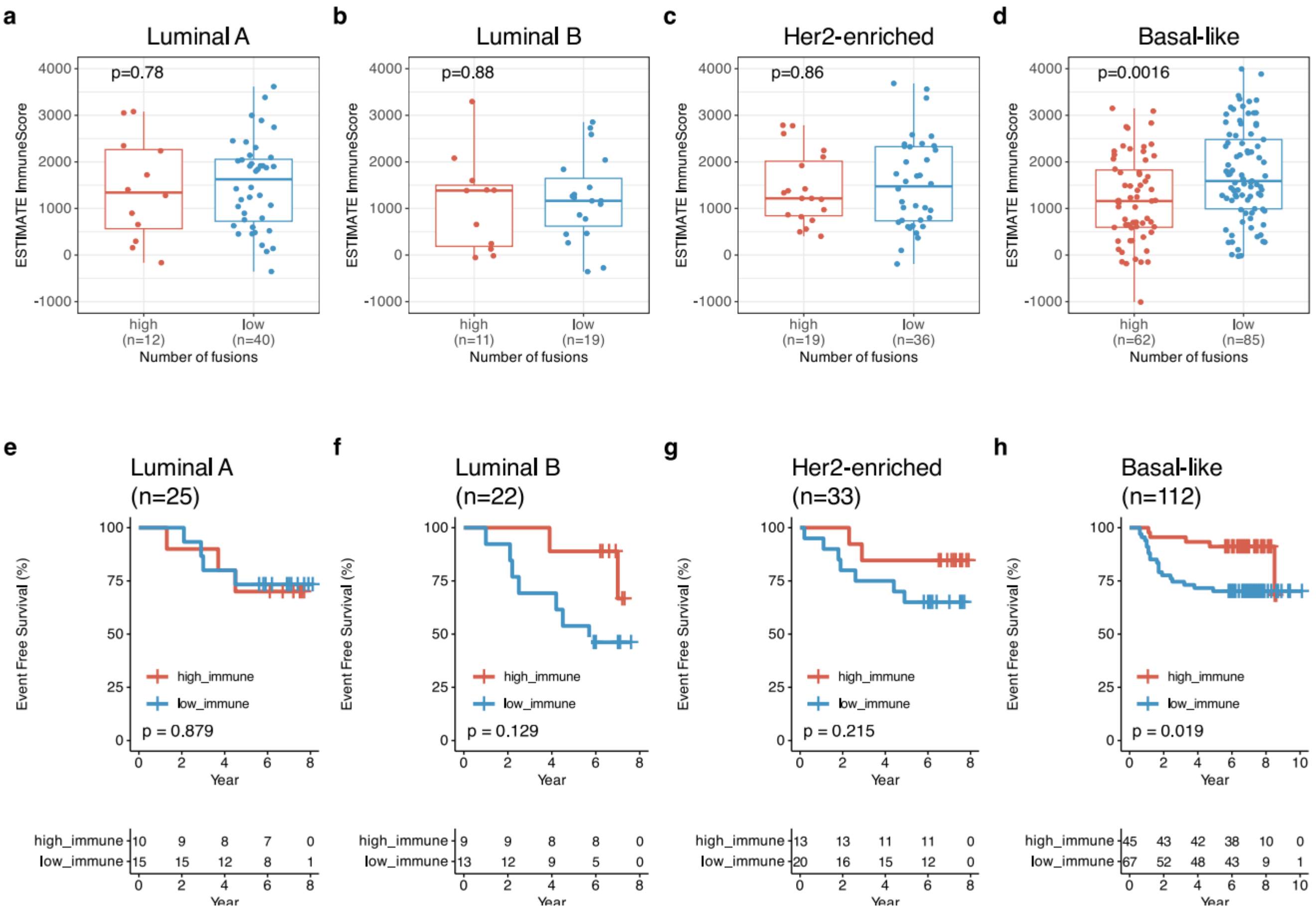

Supplementary Fig. 3: Kaplan-Meier for recurrence-free survival in FUSCC TNBC according to low vs. high number of fusions with different cut off values (n=115).

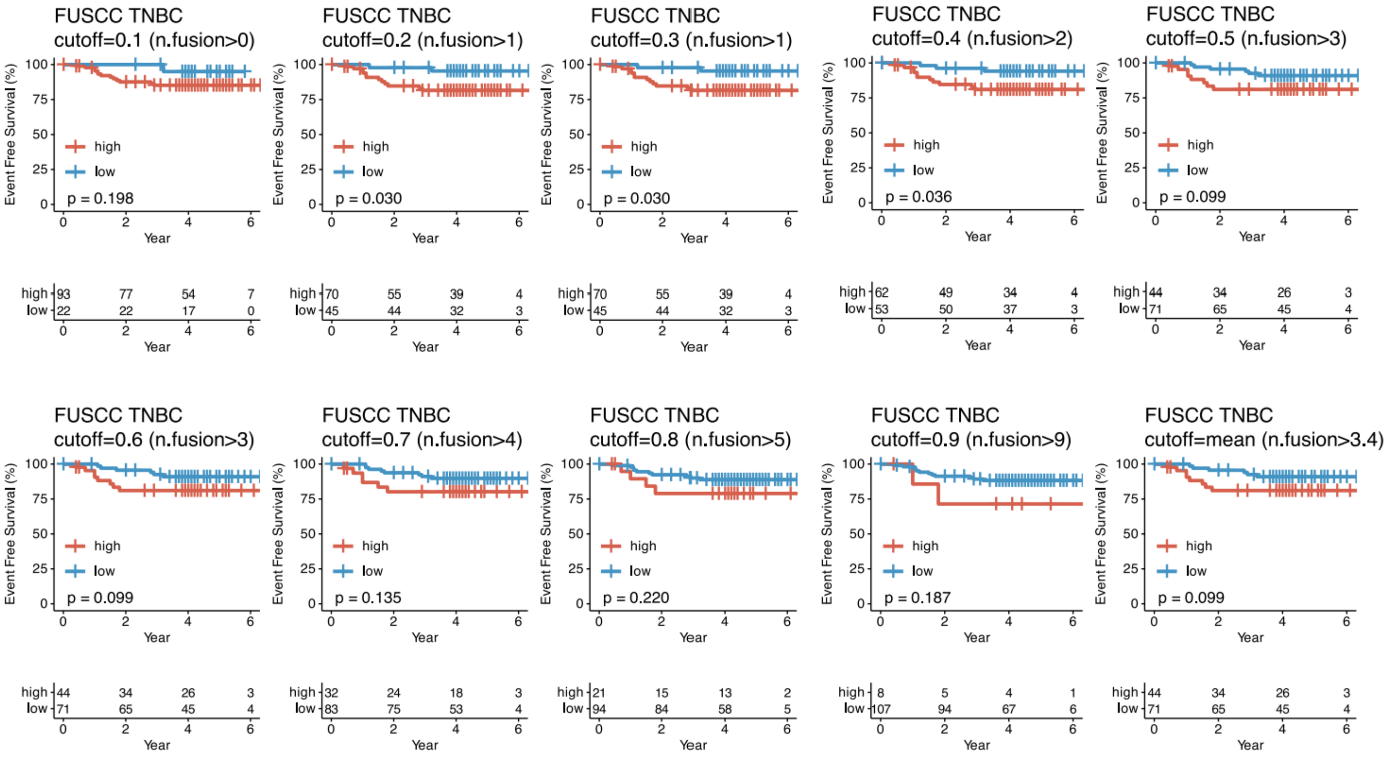

Supplementary Table 1. List of explorative studies and clinical trials analyzed in this study

Explorative / Clinical trial	WTS	WES	NCT number
Genetic alteration after systemic treatment	241	115	NCT02591966
Young breast cancer cohort younger than 40 years or pregnancy associated	56	11	NCT02597179

Number of samples. WTS: whole transcriptome sequencing, WES: whole exome sequencing

Supplementary Table 2. Number of patients by BC subtypes

	HR+HER2-	HR+HER2+	HR-HER2+	TNBC	Total
WTS	38	26	20	113	197
WTS + WES	33	23	16	54	126

Number of patients. WTS: whole transcriptome sequencing, WES: whole exome sequencing

Supplementary Table 3. Number of patients by intrinsic subtypes

	Luminal A	Luminal B	Her2-enriched	Basal-like	Normal-like	Total
WTS	25	22	33	112	5	197
WTS + WES	19	19	27	57	4	126

Number of patients. WTS: whole transcriptome sequencing, WES: whole exome sequencing

Supplementary Table 4. Number of fusions per sample according to the fusion callers after filtering

	Min	1st Q	Median	Mean	3rd Q	Max
STAR.Arriba	0	4	8	10.1	14	49
STAR.Fusion	0	4	7	8.8	13	36
STAR.SEQR	0	3	5	6.5	9	43

Number of fusion events

Supplementary Table 5. Detected fusion events per BC sample

	Min	1st Q	Median	Mean	3rd Q	Max
Before filtering	19	69	94	99.85	129	311
After filtering	0	4	9	10.27	15	49

Number of fusion events

Supplementary Table 6. Top 20 frequently detected fusion

Fusion	N	Luminal A	Luminal B	Her2-enriched	Basal-like	Normal-like	Cancer fusion
FSIP1:AC013652.1	12	7 (0.135)	5 (0.167)	0 (0)	0 (0)	0 (0)	known
ZNF326:LRRC8D	6	0 (0)	0 (0)	0 (0)	6 (0.041)	0 (0)	unknown
AGFG2:TMEM225B	4	0 (0)	0 (0)	0 (0)	4 (0.027)	0 (0)	unknown
CPSF3:SELENBP1	4	0 (0)	0 (0)	0 (0)	4 (0.027)	0 (0)	unknown
CTBP2:LARS2	4	0 (0)	0 (0)	0 (0)	4 (0.027)	0 (0)	unknown
CYP7B1:WDR72	4	0 (0)	0 (0)	0 (0)	4 (0.027)	0 (0)	unknown
DERL1:TPD52	4	0 (0)	0 (0)	0 (0)	4 (0.027)	0 (0)	unknown
ITSN1:ITIH5	4	0 (0)	0 (0)	0 (0)	4 (0.027)	0 (0)	unknown
MAP3K20:RAPGEF4	4	1 (0.019)	0 (0)	0 (0)	3 (0.02)	0 (0)	known
MOV10:CAPZA1	4	0 (0)	0 (0)	0 (0)	4 (0.027)	0 (0)	unknown
NCOR2:PARP4	4	0 (0)	0 (0)	0 (0)	4 (0.027)	0 (0)	unknown
PHLPP1:PCAT1	4	0 (0)	0 (0)	0 (0)	4 (0.027)	0 (0)	unknown
PTEN:NAALADL2	4	0 (0)	0 (0)	0 (0)	4 (0.027)	0 (0)	unknown
TAOK1:FOXN1	4	0 (0)	0 (0)	0 (0)	4 (0.027)	0 (0)	unknown
TM7SF3:VRK1	4	0 (0)	0 (0)	0 (0)	4 (0.027)	0 (0)	unknown
TTLL3:ARPC4	4	0 (0)	0 (0)	0 (0)	4 (0.027)	0 (0)	unknown
UBR4:TMCO4	4	0 (0)	0 (0)	0 (0)	4 (0.027)	0 (0)	unknown
AC010326.2:SBNO2	3	2 (0.038)	1 (0.033)	0 (0)	0 (0)	0 (0)	unknown
AC090844.2:TXNL4A	3	0 (0)	0 (0)	3 (0.055)	0 (0)	0 (0)	unknown
ACAD11:AC096564.1	3	0 (0)	0 (0)	0 (0)	3 (0.02)	0 (0)	unknown

Number of fusion events (proportion of fusion events within respective intrinsic subtypes), known cancer fusion which was known as cancer-related fusion in public fusion databases

Supplementary Table 7. Cutoff analysis for high vs. low number of fusion events with consecutive cutoff values.

cutoff.value	All	HR+HER2-	HR+HER2+	HR-HER2+	TNBC	Luminal A	Luminal B	Her2-enriched	Basal-like
0.1	2 (p=0.374)	2 (p=0.784)	4 (p=0.657)	4 (p=0.805)	3 (p=0.259)	2 (p=0.248)	3 (p=0.848)	5 (p=0.746)	3 (p=0.202)
0.2	4 (p=0.652)	3 (p=0.468)	5 (p=0.868)	6 (p=0.448)	5 (p=0.082)	2 (p=0.248)	3 (p=0.848)	6 (p=0.938)	5 (p=0.051)
0.3	6 (p=0.613)	5 (p=0.88)	7 (p=0.75)	6 (p=0.448)	6 (p=0.221)	4 (p=0.179)	7 (p=0.983)	6 (p=0.938)	7 (p=0.204)
0.4	7 (p=0.709)	7 (p=0.747)	9 (p=0.959)	9 (p=0.873)	8 (p=0.045)*	6 (p=0.045)*	7 (p=0.983)	9 (p=0.671)	8 (p=0.02)*
0.5	9 (p=0.12)	7 (p=0.747)	10 (p=0.959)	13 (p=0.901)	9 (p=0.054)	6 (p=0.045)*	8 (p=0.983)	10 (p=0.892)	10 (p=0.009)*
0.6	11 (p=0.024)*	9 (p=0.402)	11 (p=0.888)	16 (p=0.472)	11 (p=0.013)*	8 (p=0.19)	9 (p=0.837)	11 (p=0.924)	12 (p=0.003)*
0.7	14 (p=0.044)*	9 (p=0.402)	11 (p=0.888)	19 (p=0.572)	14 (p=0.073)	9 (p=0.385)	11 (p=0.34)	14 (p=0.534)	15 (p=0.002)*
0.8	17 (p=0.088)	17 (p=0.659)	17 (p=0.818)	21 (p=0.841)	16 (p=0.004)*	14 (p=0.704)	17 (p=0.963)	18 (p=0.454)	17 (p=0.041)*
0.9	22 (p=0.115)	19 (p=0.744)	19 (p=0.561)	25 (p=0.376)	22 (p=0.004)*	17 (p=0.399)	19 (p=0.83)	20 (p=0.73)	23 (p=0.011)*
Mean	10.7 (p=0.098)	9.1 (p=0.402)	10.2 (p=0.959)	13.3 (p=0.901)	10.9 (p=0.044)*	8.1 (p=0.19)	9.6 (p=0.837)	11.2 (p=0.924)	11.7 (p=0.003)*

Number of fusion events (p-value from log-rank test of Kaplan-Meier analysis), *p-value<0.05. Cutoff value 0.6 was selected significant in the most subgroup
